# Face or building superiority in peripheral vision reversed by task requirements

**DOI:** 10.2478/v10053-008-0065-5

**Published:** 2009-09-08

**Authors:** Najate Jebara, Delphine Pins, Pascal Despretz, Muriel Boucart

**Affiliations:** Laboratoire de Neurosciences Fonctionnelles et Pathologies, CNCNRS, Université Lille Nord de France, CHCHRU Lille, Lille, France

**Keywords:** peripheral vision, faces, buildings, low spatial resolution, task requirements

## Abstract

Peripheral vision has been the topic of few studies compared with central vision. Nevertheless, given that visual information covers all the visual field and that relevant information can originate from highly eccentric positions, the understanding of peripheral vision abilities for object perception seems essential. The poorer resolution of peripheral vision would first suggest that objects requiring large-scale feature integration such as buildings would be better processed than objects requiring finer analysis such as faces. Nevertheless, task requirements also determine the information (coarse or fine) necessary for a given object to be processed. We therefore investigated how task and eccentricity modulate object processing in peripheral vision. Three experiments were carried out requiring finer or coarser information processing of faces and buildings presented in central and peripheral vision. Our results showed that buildings were better judged as identical or familiar in periphery whilst faces were better categorised. We conclude that this superiority for a given stimulus in peripheral vision results (a) from the available information, which depends on the decrease of resolution with eccentricity, and (b) from the useful information, which depends on both the task and the semantic category.

## Introduction

The abilities of peripheral vision have been investigated with stimuli such as digits (Strasburger, Harvey, & Rentschler, 1991; Strasburger & Renstchler, 1996; Strasburger, Rentschler, & Harvey, 1994), letters and words (Chung, Mansfield, & Legge, 1998; Melmoth & Rovano, 2003), or faces (Makela, Nasanen, Rovamo, & Melmoth, 2001; Melmoth, Kukkonen, Makela, & Rovamo, 2000), but at small eccentricities, often below 10°. These studies attempted to equalise performances between central and peripheral vision by increasing both stimulus size as a function of cortical magnification and contrast with eccentricity. When low contrast stimuli were used, peripheral recognition remained lower than foveal recognition despite adequate size scaling.

 Given that visual information covers all the visual field, it seems useful to study peripheral vision up to large eccentricities. Nevertheless, in these conditions, object perception has been the subject of few investigations. Thorpe and collaborators (Thorpe, Gegenfurtner, Fabre-Thorpe, & Bülthoff, 2001) showed that participants were able to detect the presence of animals in photographs of natural scenes, with performance still above chance at 70° eccentricity. Naïli and collaborators (Naïli, Despretz, & Boucart, 2006) reported that observers were able to perform semantic categorisation of objects as edible or not up to 30° but not above. Moreover, Boucart and collaborators (Boucart & Naili, 2005; Boucart, Naïli, Despretz, Defoort-Dhelemmes, & Fabre-Thorpe, in press) addressed the question of implicit and explicit recognition in peripheral vision with a priming paradigm. Implicit recognition, reflected in facilitation after priming in a categorisation task (animal vs. transport), was observed at 30° eccentricity for identical and same-name objects (e.g., two different types of dogs) but was confined to identical pictures at 50° eccentricity. Explicit recognition (“Have you seen the picture before?”) was only found for an eccentricity of 30° and not above. The failure of semantic priming for same-name objects at large eccentricities suggests that access to semantic information is limited at large eccentricities, but implicit object recognition is possible, as shown by the priming effect for identical objects and the performance above chance in the study of Thorpe and collaborators (2001). These results suggest that the poorer resolution of peripheral vision would allow only large-scale feature integration. Previous studies on central vision have already shown that the useful information depends on the task (Goffaux, Jemel, Jacques, Rossion, & Schyns, 2003; Oliva & Schyns, 1997; Schyns, 1998). For instance, Schyns (1998) showed that two different categorisation tasks could require different information from a given stimulus. Indeed, judging a visual stimulus to be a “Porsche” or “Mary” requires more specific information than judging it to be a “car” or a “human face”. These different task demands could be understood in terms of finer or coarser information processing and thus as requiring higher or lower spatial frequency extraction. 

Moreover, in functional brain-imaging studies, Malach and collaborators (Hasson, Levy, Behrmann, Hendler, & Malach, 2002; Levy, Hasson, Avidan, Hendler, & Malach, 2001; Malach, Levy, & Hasson, 2002), studying peripheral vision at 16° eccentricity, suggested that different object categories might have specific eccentricity biases. Indeed, Levy and collaborators (2001) showed that faces preferentially activated the cortical representation of the central visual field, whilst buildings activated the cortical representation of the peripheral visual field. A central visual-field bias was also found for other stimuli such as letters and words (Hasson et al., 2002). Malach and collaborators (2002) argued that required resolution is an important factor in organising cortical object representations: Objects whose recognition depends on analysis of fine detail (faces, words, letters…) would activate regions associated with the cortical representation of the central visual field, whereas objects whose recognition entails large-scale feature integration (buildings) would activate regions associated with the cortical representation of the peripheral visual field. Given these results, we hypothesise that peripheral vision could be more suitable for those stimuli whose analysis is mainly based on low spatial frequencies.

The present study assessed peripheral vision abilities in object perception (buildings vs. faces) up to 60° eccentricity, in three tasks expected to require finer or coarser information processing: a repetition judgement task, a familiarity judgement task, and a categorisation task. We hypothesised that object processing in peripheral vision would result not only from the available information which depends on the decrease of resolution with eccentricity but also on the useful information which depends on both the task and the semantic category. Stimuli entailing large-scale feature integration (buildings) should be better processed in peripheral vision, but this ability would be modulated by the task demands. On the basis of prior results (Levy et al., 2001), we expected that a lower spatial resolution would suffice for a successful repetition judgement for buildings (whether the stimulus was the same as in the preceding trial or different from it) more than for faces. Whether or not a correct familiarity judgement or categorisation about faces and buildings could be based on the same kind of stimulus information is not certain. In comparison with the repetition judgement task, for example, successful familiarity judgements might be restricted to lower eccentricities for buildings, too.

## Material and general method

### Participants

Sixty healthy volunteers (26 males and 34 females, mean age 25 years, ranging from 18 to 50 years old) took part in the study. All had normal or corrected-to-normal vision. They provided written informed consent and were paid for their participation. The local ethical committee approved the experimental protocol. Volunteers were divided into four groups of 15 volunteers each. A given participant was tested at only one eccentricity (6, 20, 45, or 60°), but performed the three experiments. The presentation order of the different experiments was counterbalanced across participants.

### Stimuli

**Figure 1.**
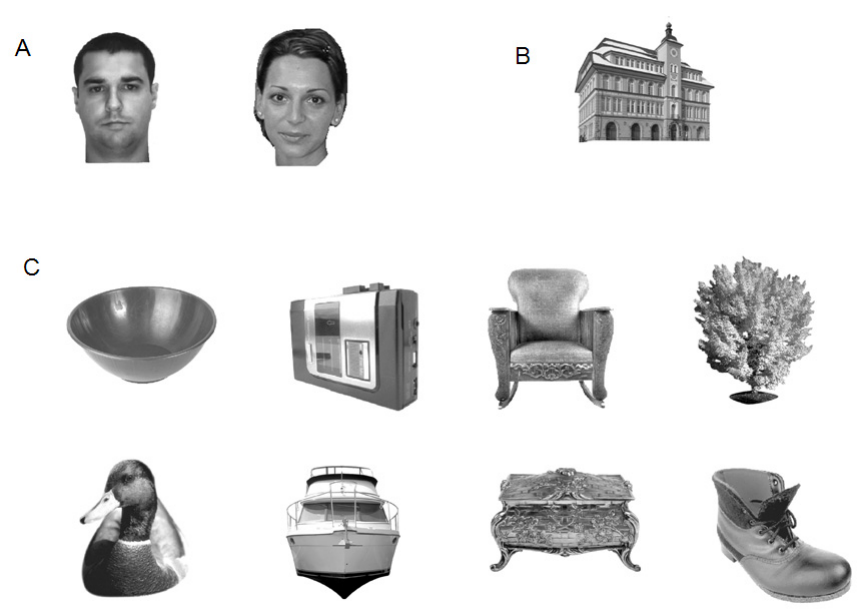
Examples of stimuli used in the different experiments for each semantic category. A: Male and female faces. B: Buildings. C: Various objects: kitchenware, high-tech, furniture, animals, vehicles, clothing, plants, and decorative objects.

All stimuli used in the three experiments were photographs (Hemera Photo Object CD-ROM library and “self-produced” photographs) belonging to three different semantic categories: male and female faces, buildings, and objects (see Figure 1). A set of 472 photographs was selected and used in the three experiments. The object category included various items: kitchenware, high-tech, furniture, animals, vehicles, clothing, plants, and decorative objects. Buildings were not considered as objects.

The study of peripheral vision required the control of the stimulus low-level characteristics. The physical characteristics of all photographs were equalised between experiments as well as between and within semantic categories. Selected photographs represented full-face objects, faces, or buildings which were isolated and presented on a white background. Excessively dark and excessively light photographs were discarded. The area covered by the different stimuli was equalised between photographs in order to use the maximum space on the images. The total image size was fixed to 591 x 591 pixels. Moreover, the original colours of each photograph used in the three experiments were converted to grey scale. Then, contrast and luminance of each selected photograph were adjusted in order to be equal in and between the different semantic categories. Thus, all photographs had a mean luminance of 16.4 cd/m^2^ (+/- 2.8 cd/m^2^) for a mean Michelson contrast of 70%. The luminance of the background was set at 60 cd/m^2^. Thus, stimuli were largely above detection threshold. The angular size of the photographs was fixed at 10° of visual angle. Since our objective was to determine the differences between central and peripheral vision and not to equalise the performance between them, the stimulus size and contrast were kept constant at each eccentricity. Once all photographs were equalised, the assignment of the photographs to the three experiments was random (except for the second task, where known stimuli were used).

Stimuli were presented at four different eccentricities in independent blocks, with their centres located respectively at 6, 20, 45, and 60°. An eccentricity of 6° was chosen to test central vision, in order to keep similar the conditions of presentation (left-right) used in the eccentricity blocks. A given photograph was only presented in one experiment to avoid stimulus repetition between experiments, but each photograph was repeated twice in each experiment.

### Apparatus and procedure

**Figure 2.**
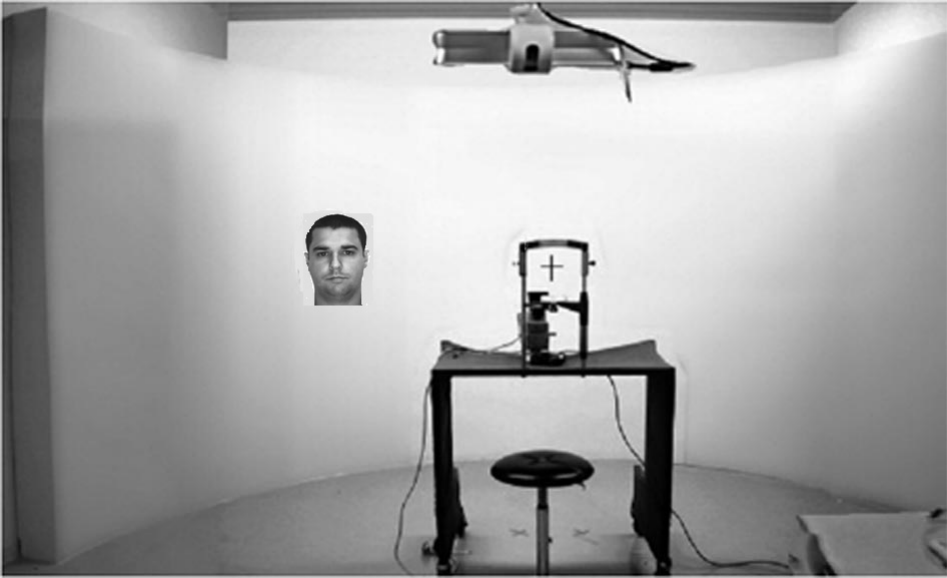
Panoramic semi-circular screen covering 180° of visual angle. The head position was stabilised by means of a chin rest. Eye movements were checked by an infrared camera.

Stimuli were presented with software developed in our laboratory (“Vision”, written by one of the authors, P. Despretz). Stimuli were displayed by means of three projectors (Sony CS5) on a panoramic semi-circular screen covering 180°. The projectors were fixed on the ceiling 3 m from the screen and connected to three graphic cards (GForce2) managed by a computer (Hewlett Packard Pentium III 1000 MHz). Participants were seated in a dark room, in front of the semi-circular screen, 2.10 m away from it (Figure 2.) A chin rest was used to stabilise head position. Participants were instructed to fixate a cross, presented during the whole experiment at the centre of the screen. Eye movements were recorded by means of an infrared camera located in front of the observer. The camera was connected to the computer and driven by the “Vision” software. When an eye movement was detected, the experiment stopped until participants looked again at the fixation cross. Photographs appeared for 100 ms at a given eccentricity. This presentation duration was short enough to avoid an exploratory saccade (180 ms on average; Rayner, 1995). A variable delay (2000 ms ±500 ms) between each photograph allowed the participants to record their response on a box containing two keys. Percentage of correct responses and response times were recorded. The experimental display is presented in Figure 2.

## Experiment 1: Repetition judgment

Experiment 1 was designed to determine whether some semantic categories were better discriminated (judged as identical or different from the previous one) than others in peripheral vision. The same repetition judgement task as the one used by Levy and collaborators (2001) in fMRI on faces and buildings was performed. The poorer resolution of peripheral vision should favour stimuli that can be discriminated on the basis of coarse information. Thus we expected to find superiority for buildings rather than faces in peripheral vision. Buildings were not considered as objects. In this experiment, objects were used as control stimuli. Indeed, this category included stimuli with very heterogeneous shapes. Consequently, they could be discriminated on the basis of coarse information, which is available in peripheral vision. If this is correct, objects should be better discriminated at all eccentricities.

### Method

For each trial, a single stimulus was randomly displayed left (50% of the trials) or right of fixation. The three semantic categories were presented in three independent blocks of 80 trials each. Forty photographs of each semantic category were used. All photographs were presented twice in a block. Half of the photographs (20) was repeated in two successive trials whilst the other half (20) was repeated at a sequential position later than the immediately succeeding trial. In each block (face, building, or object), and for each stimulus repetition (successive or not), both photographs appeared either on the same side (left or right: 50% of the trials) or on different sides (one on the left, the other on the right: 50% of the trials). The presentation order of the three trial blocks was counterbalanced across participants.

**Figure 3.**
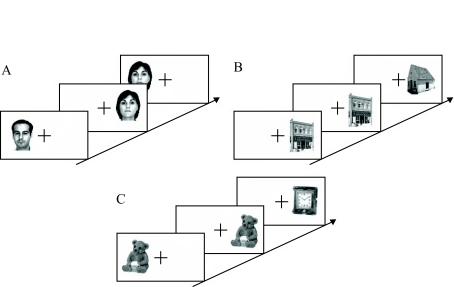
Examples of stimuli used in the repetition judgement task. Participants had to decide whether the stimulus was identical or different from the previous one, regardless of their spatial location (left-right). The different semantic categories were presented in different blocks. A: Faces. B: Buildings. C: Objects.

The task was to decide whether the displayed stimulus was identical (same photograph) or different from the previous one (see Figure 3). No answer was required for the first stimulus. Half of the participants responded “identical” with the top response key and “different” with the bottom key. The reverse stimulus-response mapping was used for the other half of the participants.

### Results

**Figure 4.**
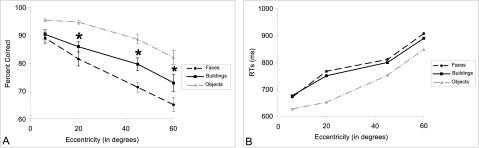
A: Percentage of correct repetition judgements. B: Response times (RTs, +/- standard errors) for objects, faces, and buildings in the repetition judgement task as a function of eccentricity. Performances were higher for buildings than for faces in peripheral vision.

Data are presented in Figure 4. ANOVAs using STATISTICA 7.0 were conducted on the percentage of correct repetition judgements (PC) and response times (RTs) including both “identical” and “different” responses, with factors of Semantic Category (object, building, and face: intra-subject variable) and Eccentricity (6, 20, 45, 60°: inter-subject variable). Trials in which eye movements were recorded were discarded (on average less than 6.5% of the trials). As our data did not respect the assumption of variance homogeneity between eccentricities, we applied an arc-sine transformation to the percentages of correct repetition judgements and a logarithmic transformation to reaction times (e.g., Howell, 1998). Levene’s test (STATISTICA 7) was applied to the data to check the variance homogeneity after transformations; PC: faces, *F*(3, 56) = 2.6, *ns*; buildings, *F*(3, 56) = 1, *ns*; RTs: faces, *F* < 1, *ns*; buildings, *F*(3, 56) = 2.7, *ns*. The variance homogeneity between eccentricities was restored. Therefore, conditions for performing an ANOVA were attained.

Objects were easier to discriminate than faces and buildings in both central and peripheral vision; main effect, PC: *F*(1, 56) = 180.4, *p* < .001; RTs: *F*(1, 56) = 34.2, *p* < .001. Only faces and buildings were taken into account for further analyses. Performance decreased significantly with the increase in eccentricity; 6°: 89.7% and 674 ms vs. 60°: 69% and 897.8 ms; PC: *F*(3, 56) = 20.8, *p* < .001; RTs: *F*(3, 56) = 10.9,

*p* < .001. A significant effect of semantic category (face and building) was observed for accuracy; *F*(1, 56) = 31.8, *p* < .001; with a better performance for buildings (82.2%) than for faces (76.8%). This effect did not reach statistical significance for RTs; *F*(1, 56) < 1, *ns*. No significant interaction between eccentricity and semantic category was observed either for accuracy, *F*(3, 56) = 2.5, *p* < .08; or for RTs, *F*(3, 56) < 1, *ns*. Nevertheless, as can be seen in Figure 4, whilst no significant difference between the two semantic categories was observed in central vision; PC 6°: *F*(1, 56) < 1, *ns*; accuracy was significantly higher for buildings than for faces in peripheral vision; PC 20°: *F*(1, 56) = 5.9, *p* < .05; 45°: *F*(1, 56) = 18.8, p < .001; 60°: *F*(1, 56) = 13.9, *p* < .001. In fact, the difference between faces and buildings increased from centre to periphery up to 45° and remained stable above 45°. Nevertheless, even at 60° eccentricity, performance was still above chance for the three semantic categories; faces: *t*(14) = 5.75, *p* < .001; buildings: *t*(14) = 7.5, *p* < .001; objects: *t*(14) = 12.9, *p* < .001. Additional analyses showed that the repetition judgement was more difficult for faces and buildings when successive stimuli appeared on different sides (left-right) than on the same side, *F*(1, 56) = 86.6, *p* < .001.

### Discussion

The main objectives of Experiment 1 were, first, to evaluate our perceptive ability in peripheral vision in a repetition judgement task and, second, to determine whether some semantic categories were better discriminated than others. Such a task was supposed to involve detailed analysis of faces, whilst large-scale feature integration should be sufficient for buildings (Levy et al., 2001). We therefore expected that buildings would be better discriminated than faces in peripheral vision.

Whatever the eccentricity, performance for objects was better than for the other two semantic categories (faces and buildings). In fact, the object category included various items (see Figure 1). Their more heterogeneous shapes could be responsible for the difference observed in performance compared with faces and buildings which constitute more homogeneous categories. For both faces and buildings, performance decreased with eccentricity: Accuracy decreased and response times increased with eccentricity. This can be explained by the decrease of available information in peripheral vision (e.g., Büser & Imbert, 1987). Nevertheless, such a repetition judgement task, even if easier in central vision, can be performed up to 60° eccentricity. Indeed, for both faces and buildings, performances remained above chance level, even at 60° eccentricity with 69% correct responses on average. Thus information available in peripheral vision still allows discriminating between two faces or two buildings.

Whilst no difference in performance between the two semantic categories (faces and buildings) was found in central vision, a superiority was found for buildings in peripheral vision (from 20 to 60°). The equivalent performance found in central vision, where all stimulus information is available, indicates that the two series of photographs were equivalent in discriminability. Moreover, the fact that objects showed higher performance than the two other semantic categories, at all eccentricities, suggests that a ceiling effect cannot be responsible for the equivalent performance found in central vision for buildings and faces. Therefore, the difference observed at large eccentricities seems to be genuinely the result of peripheral vision abilities. Access to low spatial resolution information is sufficient to judge a building as identical whereas a repetition judgement for faces should involve finer details (higher spatial resolution) which are not available in peripheral vision. In central vision, the contribution of spatial frequency band-width to face processing varies across studies. Nevertheless, in recognition (Collin, Liu, Troje, McMullen, & Chaudhuri, 2004; Costen, Parker, & Craw, 1994, 1996; Parker & Costen, 1999), identification (Fiorentini, Maffei, & Sandini, 1983) and in some categorisation tasks (e.g., expression categorisation: expressive vs. neutral; Schyns & Oliva, 1999), the authors showed that face processing was best supported by high or intermediate spatial frequency information.

The results of the present study suggest that a repetition judgement for faces requires fine-detail analysis which becomes less and less available with increasing eccentricity. Moreover, it has been shown in central vision that spatial frequency content could differentially affect the processing of objects belonging to different semantic categories (Gold, Bennett, & Sekuler, 1999; Vannucci, Pia Viggiano, & Argenti, 2001). In our study, spatial frequency content was not manipulated per se but peripheral vision changed the spatial frequency information that can be used. Our results are consistent with data in functional cerebral imaging (Hasson et al., 2002; Levy et al., 2001; Malach et al., 2002) which suggested that objects associated with the cortical representation of the central visual field like faces require analysis of fine details, whereas objects associated with more peripheral cortical representations like buildings entail large-scale feature integration. That would explain why buildings can be better discriminated than faces in peripheral vision where only low spatial resolution information is available.

We conclude that there is a superiority for buildings compared with faces in peripheral vision, at least in a repetition judgement task. In fact, this superiority does not depend on the semantic content of the stimulation per se but on the physical features useful for the task. This is supported by additional analyses showing that, for both faces and buildings, repetition judgement was easier when both successive stimuli appeared on the same side, allowing a physical matching between them. This experiment also shows that the repetition judgement task can be performed at large eccentricities for both faces and buildings. Now, what happens in a task requiring more detailed analysis?

## Experiment 2: Familiarity judgement

Experiment 2 was designed to determine whether the superiority found for buildings in peripheral vision compared with faces in Experiment 1 was also found in a task requiring a judgement of familiarity. Compared with the repetition judgement task, this task can be assumed to require finer details, especially allowing some identification of the picture. Indeed, to decide if a face or a building is known or not, it is necessary to recognise them. We supposed that face recognition which requires analysis of fine details will be more difficult in peripheral vision, whilst building recognition can still be performed on the basis of low spatial frequency analysis. We expected a superiority for buildings rather than faces in peripheral vision in the familiarity judgement task.

### Method

#### Stimuli

This experiment included 56 photographs of faces and buildings. For each semantic category, half of the stimuli were faces of celebrities or famous buildings (known), the other half were unknown faces or buildings. A pilot experiment allowed us to select the stimuli. Fourteen observers, different from those involved in the main study, saw 176 photographs of known and unknown buildings and faces randomly presented. They had first to decide, for each photograph, whether the building (or the face) was known or unknown and, second, if known, to name it. Only photographs identified by more than 80% of the participants of the pilot experiment were used as known stimuli in Experiment 2.

#### Procedure and Design

**Figure 5.**
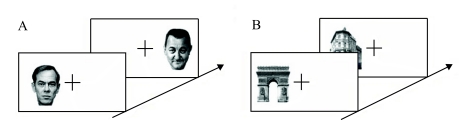
Examples of stimuli used in the familiarity judgement task. Participants had to decide whether the stimulus was known or unknown. The different semantic categories were presented in different blocks. A: Faces (the first face is unknown and the second was a French celebrity, Coluche). B: Buildings (the first building is a historic monument in Paris, l’Arc de Triomphe, and the second is unknown).

For each trial, a single stimulus was randomly displayed left (50% of the trials) or right of fixation (see Figure 5). For each semantic category, half of the “known” and “unknown” photographs appeared on the left side, the other half on the right side. The experiment was divided into two blocks of 112 trials each. In one block, faces were displayed. In the other, buildings were displayed. Each photograph was presented twice in one block. The presentation order of the two conditions was counterbalanced across participants.

The task was to decide whether the displayed stimulus was known (celebrity or famous buildings, according to the condition) or unknown. Half of the participants responded “known” with the top response key and “unknown” with the bottom key. The reverse stimulus-response mapping was used for the other half.

### Results

**Figure 6.**
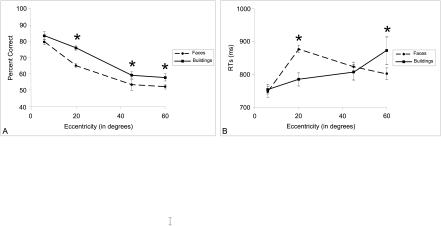
A: Percentage of correct familiarity judgements. B: Response times (RTs, +/- standard errors) for faces and buildings in the familiarity judgement task as a function of eccentricity. Performances were higher for buildings than for faces in peripheral vision.

Data are presented in Figure 6. ANOVAs using STATISTICA 7.0 were conducted on the percentage of correct familiarity judgements (PC) and response times (RTs), including both “known” and “unknown” responses, with factors of Semantic Category (building and face: intra-subject variable) and Eccentricity (6, 20, 45, 60°: inter-subject variable). Trials in which eye movements were recorded were discarded (less than 9.8% of the trials). As our data did not respect the assumption of variance homogeneity between eccentricities, the transformations used in Experiment 1 were applied to these new data. Levene’s test (STATISTICA 7) showed that, after transformations, the variance homogeneity between eccentricities was restored; PC: faces, *F*(3, 56) = 2.5, *ns*; buildings, *F*(3, 56) = 2.7, *ns*; RTs: faces, *F*(3, 56) = 1.4, *ns*; buildings, *F* < 1, *ns*. Therefore, conditions for performing an ANOVA were attained.

Performance decreased significantly with the increase in eccentricity for accuracy; 6°: 81.5%, 60°: 53.9%; *F*(3, 56) = 107.3, *p* < .001. A significant main effect of semantic category was observed for accuracy; *F*(1, 56) = 32.3, *p* < .001; with a better accuracy observed for buildings (68.5%) than for faces (61.7%). Neither of these two effects reached significance for RTs; eccentricity: *F*(3, 56) < 1, *ns*; semantic category: *F*(1, 56) < 1, *ns*. No significant interaction between eccentricity and semantic category was observed for accuracy, *F*(3, 56) < 1, *ns*. Nevertheless, although no significant difference between the two semantic categories was observed in central vision; PC, 6°: *F*(1, 56) = 3.8, *ns*; performance was significantly higher for buildings than for faces in peripheral vision, at 20° eccentricity; PC: *F*(1, 56) = 18.0, *p* < .001; RTs: *F*(1, 56) = 6.6, *p* < .05. It was easier to do a judgement of familiarity for buildings than for faces at 20° eccentricity. Performance decreased more for faces than for buildings between 6 and 20° eccentricity. Results were less clear for higher eccentricities. Indeed, as the difference between the two semantic categories remains significant for accuracy; 45°: *F*(1, 56) = 11.0, *p* < .05; 60°: *F*(1, 56) = 4.2, p < .05; this difference disappeared at 45° for RTs; *F*(1, 56) < 1, *ns*; and was actually inverted at 60° where faces gave rise to shorter RTs than buildings; *F*(1, 56) = 6.2, *p* < .05. In fact, buildings were always recognised above chance; 6°: *t*(14) = 13.4, p < .001; 20°: *t*(14) = 18.0, *p* < .001; 45°: *t*(14) = 4.3, *p* < .001; 60°: *t*(14) = 3.4, *p* < .01; whilst faces did not differ from chance at 45° eccentricity and above, 6°: *t*(14) = 17.8, *p* < .001; 20°: *t*(14) = 11.3, *p* < .001; 45°: *t*(14) < 1, *ns*; 60°: *t*(14) = 1.6, *ns*. As participants were not able to do a familiarity judgement on faces, they gave quick random responses. Therefore, RTs decreased for this category.

### Discussion

The main objectives of Experiment 2 were, first, to evaluate our perceptive ability in peripheral vision in a task involving a judgement of familiarity and, second, to determine whether the building superiority showed in a repetition judgement task was still found in a recognition task requiring finer analysis.

Once again, results showed a decrease in performance with an increase in eccentricity for both faces and buildings. The task becomes more and more difficult with increasing eccentricity for both categories of stimuli. Nevertheless, whereas stimuli can be discriminated up to 60° eccentricity, information needed to perform the familiarity judgement task was available only for buildings at 60° eccentricity, with an accuracy of 57.8% on average, but not for faces. Performance did not differ from chance for faces at 45° eccentricity and above. Familiarity judgement on faces could be performed accurately only from centre to 20° eccentricity. Nevertheless, perceptive abilities of peripheral vision are still efficient for some classes of stimuli. Indeed, whereas no difference in performance was found between faces and buildings in central vision, a superiority for buildings compared with faces was found in peripheral vision (from 20 to 60°). Once again, the difference observed in peripheral vision cannot be attributed to greater difficulty in processing one of the two series of photographs as performance was equivalent for the two categories in central vision where all information is available.

Our results suggest that familiarity judgement requires finer-detail analysis for face processing. These results are consistent with previous studies showing that face recognition and identification require high or intermediate spatial resolution (Collin et al., 2004; Costen et al., 1994, 1996; Fiorentini et al., 1983; Parker & Costen, 1999) in contrast with other tasks such as gender or expressiveness (happy/angry) categorisation (Goffaux et al., 2003; Schyns & Oliva, 1999) or detection (Halit, De Haan, Schyns, & Johnson, 2006). Hence, whereas familiarity judgement would be based on detailed analyses for faces, the global configuration, conveyed by low spatial frequencies would still be useful for the processing of buildings. Thus a familiarity judgement task can be performed on the basis of low spatial resolution information for some semantic categories. That would explain why buildings can be better recognised than faces in peripheral vision where only low spatial resolution information can still be available. Thus, our results are consistent with data in functional cerebral imaging (Hasson et al., 2002; Levy et al., 2001; Malach et al., 2002), which suggested that objects associated with the cortical representation of the peripheral visual field like buildings entail large-scale feature integration. Response times did not increase systematically with eccentricity as observed in the repetition judgement task (Experiment 1). Buildings were recognised faster than faces at all eccentricities except at 60°. Indeed, to be judged as familiar or not, faces need the processing of finer information than is available at this eccentricity. Thus at 60° eccentricity, participants were no longer able to give a judgement of familiarity on faces, giving random answers (performance does not differ from chance level), which can be done very quickly, leading to a decrease in RTs. Nevertheless, such a familiarity judgement can still be done at the same eccentricity on buildings for which coarser information is used. Indeed, even if the task becomes more and more difficult, buildings can still be recognised above chance at 60° eccentricity, leading to an increase in RTs. This study agrees in part with the work of Boucart and collaborators (Boucart & Naïli, 2005; Boucart et al., in press), showing that semantic information cannot be accessed at large eccentricities (50°), but only implicit object recognition is possible. This lack of access to semantic information would only be true for some specific semantic categories such as faces but not for others such as buildings.

From these results, we infer a superiority of buildings compared with faces in peripheral vision in both familiarity and repetition judgement tasks. Face recognition was not possible beyond 20° eccentricity. Once again, this superiority seems to depend more on the physical features which can be useful for the task than on the semantic content of the stimulus. Such a conclusion can only be confirmed by comparing the performance of these two experiments with those of a task that requires coarser information processing for both semantic categories.

## Experiment 3: CategoriSation

Experiment 3 used a categorisation task in which participants had to detect the presence of a face or a building in three types of stimulus pairs: a face and a building, a face and an object, a building and an object. Objects were only used here as comparison stimuli. Previous studies on peripheral vision (Boucart & Naïli, 2005; Boucart et al., in press; Thorpe et al., 2001) have suggested that whereas recognition is confined to small eccentricities, categorisation can still be performed at large eccentricities. The poorer resolution of peripheral vision should allow to do categorisation tasks if they require only large-scale feature integration. We therefore tested whether performance could indeed be higher for both buildings and faces. Nevertheless, all faces share a similar global shape whereas buildings are more heterogeneous in shape. Therefore it can be hypothesised that face categorisation, unlike building categorisation, can be performed on coarser information conveyed by low spatial frequencies.

### Method

**Figure 7.**
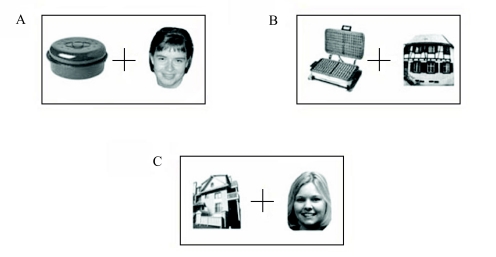
Examples of stimulus pairs used in the categorisation task. A: Object and face. B: Object and building. C: Building and face. Observers had to decide if one of the two stimuli was a face or a building according to the condition.

For each trial, two stimuli were displayed simultaneously, left (50% of the trials) and right of fixation. Eighty photographs of each semantic category were used. Three types of stimulus pairs were used (see Figure 7): a face and a building, a face and an object, a building and an object. Thus, faces and buildings were present in two thirds of the trials. For each type of pair, each semantic category appeared as many times on the left as on the right. The three types of pairs were randomly presented from one trial to another and the presentation order of the different pairs of stimuli was counterbalanced across participants.

The experiment was divided into two blocks of 120 trials each. Each photograph was displayed twice in one block, but differed from one block to the other. Forty trials of each type of stimulus pair were presented in each block. All pairs were different. The task was to decide whether one of the two stimuli displayed simultaneously was a face or a building, according to the condition. Half of the participants responded “face” or “building” (according to the condition) with the top response key and “no face” or “no building” with the bottom key. The reverse stimulus-response mapping was used for the other half of the participants. The presentation order of the two blocks was counterbalanced across participants.

### Results

**Figure 8.**
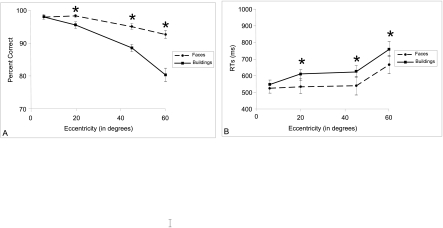
A: Percentage of correct categorisation. B: Response times (RTs, +/- standard errors) for faces and buildings in the categorisation task as a function of eccentricity. Performances were higher for faces than for buildings in peripheral vision.

Data are presented in Figure 8. ANOVAs using STATISTICA 7.0 were conducted on the percentage of correct categorisation (PC) and RTs including both “face” or “building” and “no face” or “no building” responses, with the same factors as in Experiment 2. Trials in which eye movements were recorded were discarded (on average less than 7.3% of the trials). As our data did not respect the assumption of variance homogeneity between eccentricities, the transformations used in the two previous experiments were applied to these new data. Levene’s test (STATISTICA 7) showed that after transformations the variance homogeneity between eccentricities was restored; PC: faces, *F* < 1, *ns*; buildings, *F*(3, 56) = 1.3, *ns*; RTs: faces, *F*(3, 56) = 1.6, *ns*; buildings, *F*(3, 56) = 1.7, *ns*. Therefore, conditions for performing an ANOVA were attained.

Performance decreased significantly with the increase in eccentricity; 6°: 97.9% and 536.7 ms vs. 60°: 86.4% and 716.4 ms; PC: *F*(3, 56) = 37.4, *p* < .001; RTs: *F*(3, 56) = 20.9, *p* < .001. A significant effect of semantic category was observed; PC: *F*(1, 56) = 65, p < .001; RTs: *F*(1, 56) = 82, *p* < .001; with a better performance for faces (PC = 95.9%, RTs = 565.8 ms) than for buildings (PC = 90.5%, RTs = 635.8 ms). A significant interaction between eccentricity and semantic category was observed for both accuracy, *F*(3, 56) = 7.8, *p* < .001; and RTs, *F*(3, 56) = 3.7, *p* < .05. Indeed, performance decreased more for buildings than for faces with the increase in eccentricity. As can be seen from Figure 8, the difference in performance between the two semantic categories (faces and buildings) increased with eccentricity (difference in PC: from 0.1% at 6° to 12% at 60° eccentricity; difference in RTs: from 23.8 ms at 6° to 94.2 ms at 60° eccentricity). Whereas no significant difference between the two semantic categories was observed in central vision; PC: *F*(1, 56) < 1, *ns*; RTs: *F*(1, 56) = 1.3, *ns*; performance was significantly better for faces than for buildings in peripheral vision; PC, 20°: *F*(1, 56) = 11.0, *p* < .01; 45°: *F*(1, 56) = 25.2, *p* < .001; 60°: *F*(1, 56) = 51.9, *p* < .001; RTs, 20°: *F*(1, 56) = 10.4, *p* < .01; 45°: *F*(1, 56) = 12.6, *p* < .001; 60°: *F*(1, 56) = 7.4, p < .01.

**Figure 9.**
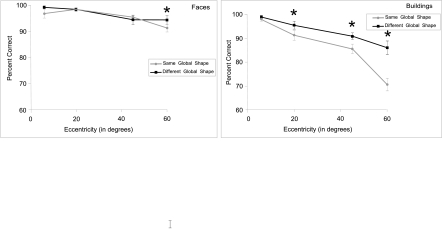
Percentage of correct categorisation as a function of eccentricity for faces compared with objects with identical (round) or different (angular) shapes and for buildings compared with objects with identical (angular) or different (round) shapes. At large eccentricities, performances were lower when faces or buildings had to be compared with objects with similar global shape.

Faces have round shapes, whereas buildings tend to have angular shapes with straight lines and angles. Additional analyses (see Figure 9) showed that the categorisation was more difficult when both stimuli in a pair had the same global shape (either angular or round) compared with stimuli which had different global shapes, *F*(1, 56) = 33.0, p < .001. Thus, when faces were presented in pairs with round objects (e.g., apple) rather than with angular objects, it was more difficult to categorise faces, *F*(1, 56) = 7.1, *p* < .05. In the same way, when buildings were presented in pairs with angular objects rather than with round objects, it was more difficult to categorise buildings; *F*(1, 56) = 22.3, p < .001. A significant interaction between shape similarity and eccentricity was observed; *F*(3, 56) = 3.6, *p* < .05. This interaction was only significant for buildings; *F*(3, 56) = 2.8, *p* < .05. Whereas no significant effect of shape similarity was observed in central vision for buildings; *F*(1, 56) < 1, *ns*; accuracy was significantly higher when buildings were compared with round objects than with angular objects in peripheral vision; 20°: *F*(1, 56) = 4.2, *p* < .05; 45°: *F*(1, 56) = 4.0, *p* < .05; 60°: *F*(1, 56) = 22.0, *p* < .001. For faces, the shape similarity effect was only significant at 60° eccentricity: Accuracy was higher when faces were compared with angular objects rather than with round objects at 60°; *F*(1, 56) = 6.8, *p* < .05. Moreover, even when both stimuli in a pair had the same global shape, faces were significantly better categorised than buildings in peripheral vision; 6°: *F*(1, 56) = 0.2, *ns*; 20°: *F*(1, 56) = 17.2, *p* < .001; 45°: *F*(1, 56) = 19.6, *p* < .001; 60°: *F*(1, 56) = 40.9, *p* < .001.

### Discussion

The main objectives of Experiment 3 were, first, to confirm the perceptive abilities of peripheral vision in a categorisation task and, second, to determine whether the superiority observed for a given semantic category differed with task requirements. Compared with the two previous tasks, the categorisation was assumed to involve, even for face processing, large-scale feature integration and therefore to be less vulnerable to the poorer resolution of peripheral vision. We expected that the task requirements might interfere with the superiority observed for buildings in peripheral vision in the previous tasks.

The results showed, as in Experiments 1 and 2, a decrease in performance with increasing eccentricity for both faces and buildings. The task becomes more and more difficult with increasing eccentricity whatever the stimulus to process. Moreover, as suggested by previous studies on peripheral vision (Boucart & Naïli, 2005; Boucart et al., in press; Thorpe et al., 2001), such a categorisation task, even if easier in central vision, can be performed at 60° eccentricity. Indeed, for both faces and buildings, performance was broadly above chance, even at 60° eccentricity, with 86.4% correct responses on average. Thus, the information available in peripheral vision still allows the categorisation of faces and buildings.

Whereas no difference in performance was found between faces and buildings in central vision, a superiority for faces compared with buildings was found in peripheral vision (from 20 to 60°). Once again, the difference observed in peripheral vision cannot be attributed to greater difficulty in processing one of the two series of photographs, as performance was equivalent in central vision where all information is available. The interaction between eccentricity and semantic category showed that the difference in performance between the two semantic categories (faces and buildings) increased with eccentricity. Performance decreased more for buildings than for faces.

Although this categorisation task can be performed on the basis of low spatial resolution for both buildings and faces, our results suggest that it requires finer-detail analysis for the processing of buildings than for faces. That would explain why faces can be better categorised than buildings in peripheral vision where only low spatial resolution information is available. In fact, faces are more structurally homogeneous than buildings. They have a specific round shape and share the same spatial configuration (two eyes above a nose above a mouth). This specificity of faces compared with buildings allows faces to be more easily categorised among various stimuli. Buildings have more varied shapes, and they can be confused with other objects. This interpretation is strengthened by additional analyses showing that categorisation was easier in peripheral vision when faces and buildings had to be compared with objects with a different global shape than with objects with a similar global shape.

We conclude that, contrary to the two previous experiments, there is a superiority for faces in peripheral vision compared with buildings in such a categorisation task. This confirms that the superiority seems to depend more on physical features which are useful for the task than on the semantic content of the stimulus.

## Comparison of tasks

ANOVAs were conducted on the percentage of correct responses (PC) and RTs, with factors of Semantic Category (building and face: intra-subject variable), Eccentricity (6, 20, 45, 60°: inter-subject variable) and Task (repetition judgement, familiarity judgement, and categorisation: Intra-subject variable).

Performance decreased significantly with the increase in eccentricity for both accuracy; *F*(3, 56) = 73.5, *p* < .001; and RTs, *F*(3, 56) = 7.2, *p* < .001. The main effect of task was significant for both accuracy; *F*(2, 112) = 418.6, *p* < .001; and RTs; *F*(2, 112) = 169.8, *p* < .001; with a better performance for the categorisation task (Experiment 3: 93.2% and 600.9 ms) than for the repetition judgement task (Experiment 1: 79.5% and 783.8 ms) and for the familiarity judgement task (Experiment 2: 65.4% and 808.6 ms). A significant interaction between task and eccentricity was found for both accuracy; *F*(6, 112) = 2.5, *p* < .05; and RTs; *F*(6, 112) = 5.4, *p* < .001. Performance decreased more with the increase in eccentricity for the familiarity judgement task (Experiment 2) followed by the repetition judgement task (Experiment 1) and by the categorisation task (Experiment 3): The lower the performance in central vision, the larger the decrease in performance with eccentricity. A significant interaction between task, semantic category, and eccentricity was found for both accuracy; *F*(6, 112) = 5.2, *p* < .001; and RTs; *F*(6, 112) = 3.1, *p* < .01. Performance was better in peripheral vision for buildings than for faces in the repetition judgement and the familiarity judgement tasks (Experiments 1 and 2) whereas it was better for faces than for buildings in the categorisation task (Experiment 3).

## General Discussion

One of the main results of this study is the superiority found for a specific semantic category in peripheral vision. Nevertheless, this superiority depends on the task requirements. Indeed, a difference in performance between buildings and faces was found in peripheral vision only. This difference did not appear in central vision where both semantic categories led to equivalent performance, revealing that there is no bias between the different types of stimuli used. Thus, these results suggest that in central vision, whatever the stimulus, all the information required by the different tasks is available and can be processed. On the other hand, the information available in peripheral vision does not allow an equivalent processing for the different stimuli. Peripheral vision shows a graduate decrease in spatial resolution accounting for the decrease of performance with eccentricity. The superiority found for some semantic categories was observed at eccentricities as great as 60°, suggesting that in peripheral vision a given stimulus can be better processed than another simply because of its content in low spatial frequencies. Studies on central vision have already shown that each semantic category requires different involvement of low and high spatial frequency channels. Indeed, using spatial frequency filtering to investigate the information required for stimulus processing, these studies showed that face recognition was best supported by an intermediate spatial frequency range (Collin et al., 2004; Costen et al., 1994, 1996; Fiorentini et al., 1983; Parker & Costen, 1999), whereas letters could be identified over a wider range of spatial frequencies (Gold et al., 1999). Vannucci and collaborators (2001) showed that animals were identified with a lower resolution level than non-living objects whereas vegetables needed an intermediate resolution level. Thus, the superiority observed in peripheral vision for specific semantic categories results from processing based on a selective low spatial frequency range.

Previous brain-imaging studies on object perception in peripheral vision have shown a peripheral bias for objects as buildings compared with faces (Hasson et al., 2002; Levy et al., 2001; Malach et al., 2002), suggesting that the processing of building entailed large-scale feature integration. Our results are consistent with this assumption in Experiments 1 and 2 where a superiority for buildings compared with faces was found in peripheral vision. In these tasks, the processing of buildings could be based partly on their low spatial frequency content. Thus, for tasks like repetition judgement or familiarity judgement, known to require finer-detail analysis, some stimuli (such as buildings) can be better processed than others (such as faces) in peripheral vision on the basis of their low spatial resolution content. In contrast, face processing seems to require higher spatial frequency information to be discriminated or judged as familiar. These results are consistent with studies in central vision showing that face recognition or identification can be based on intermediate or high spatial resolution (Collin et al., 2004; Costen et al., 1994, 1996; Fiorentini et al., 1983; Parker & Costen, 1999). Nevertheless, the superiority observed for buildings was not found in peripheral vision in Experiment 3 where a categorisation task was used. On the contrary, we showed a superiority for faces compared with buildings. Face categorisation would be facilitated by their more specific and homogeneous configuration. Thus, faces could not be confounded with objects of other semantic categories. This interpretation is consistent with the study of Rousselet and collaborators (Rousselet, Macé, & Fabre-Thorpe, 2003), suggesting that faces constitute a special object class which is automatically detected and segregated by our visual system. Therefore face categorisation, based on the global configuration of the stimuli, would depend more on low spatial resolution. Studies in central vision have already shown a modulation of the spatial frequency range used as a function of task requirements (Goffaux et al., 2003; Morrison & Schyns, 2001, for a review; Oliva & Schyns, 1997; Schyns, 1998; Schyns & Oliva, 1999). In peripheral vision, the superiority observed for one or the other semantic categories would then be modulated by the task being performed. Indeed, a given task can require a simple global shape analysis for a stimulus and finer-detail analysis for another one. The relevant spatial frequency range used to process an object in peripheral vision depends not only on the semantic category but also on the specific requirement of the task.

The different tasks used do not present the same level of difficulty. Indeed, the categorisation of a given object seems to be the easiest task, whilst the familiarity judgement is more difficult than the repetition judgement. This difference between tasks increased with the increase in eccentricity. In fact, the decrease in performance with eccentricity is larger when the task is more difficult. While categorisation or repetition judgement for buildings and faces can be performed up to 60° eccentricity, familiarity judgement seems to be restricted to smaller eccentricities (below 45°) at least for faces. The difficulty, inherent in each task, seems to be reproduced at all eccentricities, but with an additional factor which increases the difference between tasks in peripheral vision. This factor is related to the general task demand in terms of spatial resolution. Whereas the available information about details or high spatial frequency decreases with eccentricity, tasks requiring high spatial resolution become more difficult. Thus, in our study, familiarity judgement involved more high spatial frequency processing than repetition judgement or categorisation tasks. Peripheral vision emphasises the difference between tasks depending on their specific spatial scale requirements.

Finally, peripheral vision, with its low resolution, still allows the processing of stimuli such as faces and buildings. The ability of peripheral vision for object categorisation, already reported in previous studies (Boucart & Naïli, 2005; Boucart et al., in press; Naïli et al., 2006; Thorpe et al., 2001), is extended here to repetition judgement and familiarity judgement. Although object perception is usually attributed to central vision because of its high spatial resolution, our results suggest that peripheral vision can be used as well. Indeed, depending on the semantic category, peripheral vision can provide access to enough information to categorise, discriminate and even give a judgement of familiarity. To conclude, our study not only shows a superiority for some specific stimuli in peripheral vision, as the study of Levy and collaborators (2001) suggests, but this superiority is modulated by the task to be performed. Thus, object perception in peripheral vision results not only from the available information which depends on the decrease of resolution with eccentricity but also on the useful information which depends on both the task and the semantic category.
